# Comparative effectiveness of digital mental healthcare models for adults with epilepsy: A study protocol of a randomized controlled trial

**DOI:** 10.1002/epi4.12913

**Published:** 2024-02-12

**Authors:** Milena Gandy, Honor Coleman, Henry Cutler, Michael P. Jones, Eyal Karin, Patrick Kwan, Armin Nikpour, Kaitlyn Parratt, Genevieve Rayner, Nickolai Titov, Lisa Todd, Elizabeth Seil, Toby Winton‐Brown, Wendy Wu, Blake F. Dear

**Affiliations:** ^1^ School of Psychological Sciences Macquarie University Sydney New South Wales Australia; ^2^ Melbourne School of Psychological Sciences The University of Melbourne Melbourne Victoria Australia; ^3^ Australian Institute of Health Innovation Macquarie University Sydney New South Wales Australia; ^4^ Macquarie University Centre for the Health Economy Sydney New South Wales Australia; ^5^ Macquarie University Business School Sydney New South Wales Australia; ^6^ Department of Neuroscience, Central Clinical School Monash University Melbourne Victoria Australia; ^7^ Department of Neurology Royal Prince Alfred Hospital Camperdown New South Wales Australia; ^8^ The Epilepsy Society of Australia South Australia Australia; ^9^ Comprehensive Epilepsy Program Alfred Hospital Melbourne Victoria Australia; ^10^ MindSpot MQ Health, Macquarie University New South Wales Australia; ^11^ Epilepsy Action Australia Sydney New South Wales Australia

**Keywords:** anxiety, cognitive behavior therapy, depression, mood, online

## Abstract

**Objective:**

Mental health complaints are prevalent among people with epilepsy, yet there are major barriers that prevent access to psychological care, including high out‐of‐pocket costs and a lack of accessible specialized services. The purpose of the current study is to examine the comparative efficacy, acceptability, cost‐effectiveness, and long‐term outcomes of a digital psychological intervention when delivered under two models of care (i.e., guided vs. unguided) in supporting the mental health and functioning of adults with epilepsy.

**Method:**

Approximately 375 participants across Australia will be enrolled. Eligible participants will have a confirmed diagnosis of epilepsy, experience difficulties with their emotional health, be at least 18 years of age, and live in Australia. Participants will be randomized (2:2:1) to receive the Wellbeing Neuro Course, a 10‐week internet‐delivered program, with (i.e., guided) or without guidance by a mental health clinician (i.e., unguided), or be allocated to a treatment‐as‐usual waiting‐list control group. Participants will complete online questionnaires at pre‐, post‐treatment, and 3‐ and 12‐month follow‐up and consent to have their data linked to their medical records to capture healthcare system resource use and costs.

**Analysis:**

Primary outcome measures will be symptoms of depression and anxiety. A cost‐utility analysis will be undertaken using the Australian healthcare system perspective and according to current economic evaluation guidelines. Resource use and costs to the healthcare system during the study period will be captured via data linkage to relevant administrative datasets in Australia.

**Significance:**

The results of this trial will provide important data concerning the relative outcomes of these different models of care and will inform the integration of digital psychological interventions translation into healthcare services.

**Ethics and Dissemination:**

The Human Research Ethics Committee of Macquarie University approved the proposed study (Reference No: 520231325151475). The results will be disseminated through peer‐reviewed publication(s).

**ANZCTR Trial Registration Number:**

ACTRN12623001327673.

**Plain Language Summary:**

This study seeks to find out if a 10‐week online psychological treatment can improve the mental health and well‐being of Australian adults with epilepsy. Around 375 participants will be randomly assigned to different groups: one will receive treatment with guidance from mental health clinician (guided group), one without guidance (unguided group), and one starting later (waiting control group). All participants will fill out the same outcome measures online. The main goal of this research is to compare these groups and assess how well the treatment works in improving mental health outcomes.

**Key points**

- Randomized controlled trial comparing two models (guided vs unguided) of delivering a digital psychological intervention, with a control.
- Adults with epilepsy living in Australia will be recruited.
- The intervention aims to improve mental health and functional outcomes, with primary outcomes being symptoms of depression and anxiety.
- The guided model gets the intervention with support from a mental health clinician, the unguided model gets the intervention alone.
- The comparative efficacy, acceptability, cost‐effectiveness, and long‐term outcomes of these models will be assessed.

## 1 INTRODUCTION

Poor mental health is highly prevalent among people with epilepsy (PWE), with one in three estimated to have a lifetime mental health diagnosis.^1^ Comorbid neurological and mental health difficulties can increase healthcare utilization and burden of disease,^2^, ^3^ with poor mental health disrupting the medical management, self‐management, quality of life, morbidity, and prognosis of epilepsy, even when symptoms are mild.^1^, ^4^, ^5^, ^6^ Despite this, there continues to be significant unmet mental health needs of PWE worldwide, prompting recent calls for reform in this area by the World Health Organization.^7^

Despite evidence that psychological treatments can lead to significant improvements in mental health and quality of life for PWE,^8^, ^9^ there are significant barriers that prevent access to such treatments. Highlighting this, a recent survey of epilepsy health professionals found that over 50% were under‐resourced to manage mental health concerns of their patients.^10^ Common barriers to care included a lack of standardized procedures to manage mental health in epilepsy settings and a lack of accessible mental health specialists and interventions.^11^, ^12^ Instead, most PWE within Australia need to arrange their own mental healthcare, and those who try, report significant barriers to accessing effective care, including high out‐of‐pocket costs, travel restrictions, and a lack of specialists and services.^13^ To complicate matters, many PWE report a lack of independence (e.g., lack of driving license) and significant difficulties with their cognitive function (e.g., inattention and poor memory), which can impede their ability to both attend and benefit from traditional face‐to‐face psychological interventions.^4^, ^14^

Digital mental health interventions (i.e., that do not require face‐to‐face contact) are an innovative development that is improving access to psychological care.^15^ These treatments can teach the same core psychological skills as face‐to‐face treatments, such as cognitive restructuring and behavioral activation, but use carefully developed online modules to facilitate learning. Thus, people can engage in these treatments at their own pace, from the convenience of their own home, and potentially at a much lower cost. Digital interventions can also be optimized for cognitive impairment as people can read and revise content as many times as needed. Substantial research supports the efficacy of digital interventions and their clinical equivalence with face‐to‐face psychological care in the general population.^16^, ^17^ There is emerging evidence for the efficacy of digital psychological interventions to improve symptoms of depression and anxiety in adults with neurological disorders including Parkinson's disease,^18^, ^19^ multiple sclerosis (MS),^20^, ^21^, ^22^ migraine,^23^ and epilepsy.^24^, ^25^

Digital mental health interventions can be provided in both guided and unguided models, each with its own potential strengths and weaknesses. In guided models, intervention materials are provided alongside patient‐centered care from a mental health clinician, usually over the phone and/or secure messaging systems. This model of care requires available trained clinicians and their costs to be covered, making the model more expensive and complicated to implement. In unguided models, participants get access to intervention materials but work through the course independently. Unguided models can either involve access to an unguided (or self‐guided) intervention following an initial interview with a clinician or a completely automated intervention with no interview or clinical contact. Research suggests that fully automatic programs experience high dropout rates compared to unguided (with interview) models.^26^ In the context of this research, unguided models refer to those that also include a clinical interview and assessment prior to participation.

Meta‐analytic evidence in the general population, and for those with chronic pain, suggests that on average guided interventions are more clinically efficacious than unguided interventions.^27^, ^28^ In contrast, unguided models have greater public health potential, as they cost less, are easier to implement in routine care settings, and have been found to be efficacious for mild/subthreshold depression.^28^ Yet, participants with severe or complex mental health presentations, such as those with neurological comorbidities including epilepsy, may not achieve good clinical outcomes without clinician support.

Thus, the comparative clinical and cost‐effectiveness of guided and unguided digital interventions remains an important topical area of investigation^15^ and critical information to inform healthcare service providers. Reflecting this, a carefully developed digital chronic pain management intervention was similarly clinically efficacious and acceptable in an unguided and guided model; however, the unguided model was more cost‐effective.^29^ This research highlights the importance of comparative effectiveness trials to inform decision‐makers about the relative merits of different models of care. One recent randomized controlled trial (RCT) in adults with MS compared the efficacy of a digital cognitive behavior‐based program in a guided or unguided format with a treatment‐as‐usual, waitlist, control group.^22^ Both versions of the program led to a significant reduction in depression symptoms compared to the control group. However, a direct comparison between the guided and unguided groups was not performed. To date, there have been two RCTs of digital psychological interventions in epilepsy, both of which utilized unguided models of care.^24^, ^25^ Notably, while both reported that the treatment arms experienced significant improvements in symptoms of depression compared to controls, they also experienced relatively high dropout rates (>25%). On the other hand, an open trial of a guided digital psychological intervention to improve mental health in PWE observed very low rates of attrition (4%).^30^ These findings highlight potential differences in intervention engagement based on levels of guidance. However, conclusions in this area can only be reached following clinical trials that directly compare these different care models.

The aim of the current study is to assess the comparative effectiveness and acceptability of a digitally delivered psychological intervention aimed at improving both mental health (e.g., depression and anxiety) and functional outcomes (e.g., day‐to‐day disability and perceived cognitive function) of Australian adults with epilepsy. Specifically, this three‐armed RCT will directly compare two clinical care models (guided vs. unguided) of delivering an established digital intervention, the Wellbeing Neuro Course, with a treatment‐as‐usual waitlist control (TAU‐WLC) group.

Consistent with previous trials, we hypothesize that:

1. Both the guided and unguided groups will result in substantial improvements in primary outcomes of depression and anxiety compared to the TAU‐WLC group.
2. There will be non‐inferiority in clinical efficacy between the guided and unguided groups across the primary outcomes.
3. Both the unguided group and guided group will be cost‐effective compared to TAU‐WLC, but the unguided group will be more cost‐effective relative to the guided group due to lower intervention costs.

## 2 METHOD

### 2.1 Registration and ethics

This is a intervention study conducted at one center: The eCentreClinic, School of Psychological Sciences, Macquarie University, Sydney, Australia. Participants will complete questionnaires at multiple time points. Up to 375 participants will be recruited, with recruitment planned between February 2024 and January 2027.

The project is funded by an NHMRC Medical Research Future Fund grant and additional funding support from Macquarie University. Ethics approval was granted by the Human Research Ethics Committee of Macquarie University (HREC: Reference No: 520231325151475). This trial is registered with the Australian and New Zealand Clinical Trials Registry (ANZCTR; ACTRN12623001327673).

### 2.2 Study design

A three‐group CONSORT‐R Compliant RCT design will be employed. Participants will be randomized to:

1. Immediate guided treatment group (*n* = 150), who receive access to the Wellbeing Neuro Course with clinician support.
2. Immediate unguided treatment group (*n* = 150), who receive access to the Wellbeing Neuro Course without clinician support.
3. TAU‐WLC group (*n* = 75), who will crossover to receive access to the Wellbeing Neuro Course at the 3‐month follow‐up period for groups 1 and 2 (approx. 6 months from baseline). They will have the choice of receiving clinician support or doing the course unguided.

A substudy involving a brief survey of the participants' referring or primary epilepsy health professionals' views on the usefulness of the intervention and care pathway will also be conducted.

### 2.3 Participant eligibility

Eligible participants will have a confirmed diagnosis of epilepsy, self‐report experiencing difficulties with their emotional health, be aged 18 years or over, and currently living in Australia.

Exclusion criteria include those who are imminently suicidal or unable to keep themselves safe, those experiencing severe cognitive difficulties with day‐to‐day memory, attention, and ability to learn basic information, those who are unable to read and understand English, or those who do not have access to the internet.

Non‐eligible applicants will be notified and strongly encouraged to speak to their primary health professional to identify local treatment options available to them. They may also be directed to the MindSpot Clinic (www.mindspot.org.au), which offers a variety of similar courses for free to all adults within Australia. A suicide risk assessment will be conducted for all participants and appropriate triaging to external community and crisis services where appropriate.

### 2.4 Recruitment

The research will be promoted via advertisements/posters in newspapers, social media (e.g., Facebook, Twitter), and service providers and organizations providing services to PWE and other neurological disorders. This includes epilepsy organizations (e.g., Epilepsy Action Australia, Epilepsy Foundation, and Epilepsy Society of Australia) and hospital clinics (e.g., neurology departments). Advertisements may include placing flyers in neurology clinics and via notifications of the research on their website or social media.

These advertisements will direct participants to the eCentreClinic website (www.ecentreclinic.org) for more information. The eCentreClinic is a specialist, not‐for‐profit, research clinic that develops and evaluates online, and workbook‐delivered treatments for a range of common mental health and chronic health conditions with the aim of increasing access to effective, evidence‐based treatment.

The recruitment procedure is as follows:

1. Interested applicants will access a description of the study and the Participant Information and Consent Form on the eCentreClinic website. They will then elect to submit an online screening assessment, where they will complete an automated screening questionnaire to measure their symptoms and clarify that they meet the inclusion criteria.
2. Potentially eligible participants will then be contacted by an eCentreClinic mental health clinician by telephone to complete a brief screening assessment and a structured clinical interview for DSM‐5 Mood and Anxiety Disorders (QuickSCID‐5; i.e., approx. 25 min). The purpose of this assessment is to ensure participants meet the inclusion criteria, have a complete understanding of the research, and have an opportunity to ask any questions they may have. It will also allow us to gather mental health diagnostic information for each participant, but this is not an inclusion criterion.
3. Eligible participants will be sent an email confirming their position on the course. The email will contain a start date and information about the procedure for the first week of the course. On the first day of the course, participants are sent their login details with detailed instructions for working through the course. Participants are also provided with the contact details of the eCentreClinic Team and are invited to contact the Team via email in the event of technical issues. Participants in the guided group will be given instructions on how to contact their clinician. Thus, it is not possible to blind participants to group allocation.

### 2.5 Participant safety and withdrawal

Participants are monitored regularly throughout treatment and all clinicians working with participants receive regular clinical supervision in which the progress of participants is reviewed. Serious adverse events will be reported to the HREC and eCentreClinic directors.

If a participant wishes to withdraw from the study once it has started, can do so at any time without having to give a reason. This process will be supervised by the Chief Investigator. Importantly, upon withdrawal, all participants will be provided with the option to contact the eCentreClinic Team to discuss their symptoms and other treatment services and all withdrawing participants will be encouraged to access additional services via their primary health professional.

### 2.6 Procedures

The Wellbeing Neuro Course will be employed as the intervention in this study and is a digital psychological treatment for adults with neurological disorders.^31^ This intervention has been carefully developed to be suitable for adults with a broad range of neurological disorders, including PWE. It uses the principles of both cognitive behavior therapy and compensatory cognitive rehabilitation to target several domains of mental health and functional disability and covers a range of skills for managing depression, anxiety, cognitive difficulties, and activity/fatigue levels. A comprehensive overview of the intervention has been previously reported.^31^ A previous RCT of the intervention in a guided format found significant benefits on measures of depression, anxiety, disability, and cognitive function in the treatment group relative to the TAU‐WLC.^32^ Participants included a mixed sample of PWE, MS, Parkinson's disease, or an acquired brain injury. Acceptability of the treatment was also high, with over 90% of participants reporting that the course was worth their time and that they would recommend it to others.

The course includes six online lessons which are provided over a 10‐week period. Participants will work through the course according to a predetermined timetable and cannot access new materials without first having read previous materials. The course also includes worksheets for each lesson, additional written resources that can be downloaded, and case stories based on previous participants which can be followed throughout the course.

Participants allocated to the guided group will have access to support from an eCentreClinic clinician as they work through the course. All clinicians will be mental health professionals and will be employed by Macquarie University or one of its entities (e.g., MQ Health). All mental health professionals will be provided with training and supervision from a senior clinical psychologist to ensure competence and safety in their practice.

Participants in the guided group will be informed that their clinician will be available for approximately 15 min each week and will contact them throughout the course via telephone or secure messaging systems, with more time available if clinically indicated. Clinicians will be available to (1) answer questions; (2) summarize content; (3) encourage skills practice and reinforce progress; (4) enquire about participants' use of the skills; and (5) normalize challenges.

#### 2.6.1 Randomization

Participants will be randomized in a ratio of 2:2:1 to guided treatment, unguided treatment, and TAU‐WLC. The rationale for the 2:2:1 randomization sequence, with smaller weighting for control participants, is related to saving recruitment resources given that previous Phase II findings demonstrate that the control group does not improve.^32^ Randomization will be performed by an independent researcher using an online randomizer (www.random.org) using permuted blocks of 10 with 2:2:1 allocation. Participants will be stratified based on referral sources. The allocation sequence will be generated prior to enrolment of the first participant and concealed from investigators until successful participant enrolment, such that the research team are unable to affect group allocation. Randomization will also be conducted such that study personnel who determine eligibility and enroll participants are blind to which treatment allocation participants will be assigned to (i.e., allocation concealment).

### 2.7 Measures

A timeline of administration for the measures used is summarized in Table 1.

**TABLE 1:** Timeline for administration of measures.

Measure	Assessment	Pre‐treatment (baseline)	Weekly during treatment	Post‐treatment (10‐week treatment)	3‐month follow‐up	12‐month follow‐up
Participant measure						
Demographics	X					
QuickSCID‐5	X					
Condition details	X					
PHQ‐9	X	X		X	X	X
PHQ‐2			X			
GAD‐7	X	X		X	X	X
GAD‐2			X			
WHODAS 2.0	X	X		X	X	X
NDDIE	X	X		X	X	X
NDDIE‐suicide item			X			
BrEASI	X	X		X	X	X
NeurQol_Cognitive function		X		X	X	X
Seizure frequency	X	X	X	X	X	X
CCSQ		X		X	X	X
TSQ				X		
ReQOL‐10		X		X	X	
EQ‐5D‐5L		X		X	X	
Use of care services (purpose‐built based on the iMTA TIC‐P)		X		X	X	

Abbreviations: BrEASI, Brief Epilepsy Anxiety Instrument; CCSQ, Compensatory Cognitive Strategies Questionnaire; GAD‐7, Generalized Anxiety Disorder – 7; iMTA, Institute for Medical Technology Assessment; NDDI‐E, Neurological Depressive Disorder Inventory‐Epilepsy; PHQ‐9, Patient Health Questionnaire‐9; QuickSCID‐5, Quick Structured Clinical Interview for DSM‐5; ReQOL‐10, Recovering Quality of Life 10‐item; TIC‐P, Treatment Inventory of Costs in Patients with Psychiatric Disorders; TSQ, Treatment Satisfaction Questionnaire.

#### 2.7.1 Psychiatric diagnostic interview

The Mood and Anxiety Disorders Modules of the Quick Structured Clinical Interview for DSM‐5 (QuickSCID‐5) will be administered via a telephone interview.^33^

The remaining data will be collected via online questionnaires using eCentreClinic and Macquarie University's software systems.

#### 2.7.2 Demographic and clinical variables

Demographic information including gender, age, education, and employment status will be collected at the recruitment stage. Seizure frequency will also be monitored over the course of the trial.

#### 2.7.3 Primary outcomes

1. Patient Health Questionnaire 9‐Item (PHQ‐9). This is a measure of the number and severity of symptoms of depression, based on the DSM‐IV criteria for major depressive disorder.^34^
2. Generalized Anxiety Disorder 7‐Item (GAD‐7). This is a measure of symptoms of anxiety, based on the DSM‐IV criteria for GAD, but is sensitive to five different anxiety disorders.^35^

#### 2.7.4 Secondary outcomes

1. World Health Organization Disability Assessment Schedule 2.0 (WHODAS‐2). This is a 12‐item measure of disability associated with living with a chronic health condition.^36^
2. The Neuro‐QoL (Cognitive Function). This is a measure of perceived difficulties in cognitive abilities (e.g., attention, memory and decision‐making, or in the application of such abilities to everyday tasks (e.g., planning and remembering)).^37^
3. Compensatory Cognitive Strategies Questionnaire (CCSQ). This is a purpose‐built measure to assess the use of compensatory cognitive strategies before and after the intervention.^32^
4. Neurological Depressive Disorders Inventory‐Epilepsy (NDDI‐E). This is an epilepsy‐specific depression screener, which does not include any items that may be confounded by seizure phenomena and common side effects of medications.^38^ This will be administered as a confirmatory depression measure.
5. Brief Epilepsy Anxiety Survey Instrument (BrEASI). This is an epilepsy‐specific anxiety screener which attempts to remove items that may be confounded by seizure phenomena.^39^ This will be administered as a confirmatory anxiety measure.

#### 2.7.5 Quality‐of‐life and resource‐use measures

1. Recovering Quality of Life 10‐item (ReQoL‐10). This is a validated patient‐reported outcome measure used to capture mental health service users' experience of recovery in quality of life.^40^
2. EQ‐5D‐5L.^41^ This is a simple, preference‐based generic measure of health status used worldwide to inform cost‐utility analyses. This will be administered for validation purposes.
3. Use of Care Services Questionnaire, a purpose‐built measure based on the Institute for Medical Technology Assessment (iMTA) Treatment Inventory of Costs in Psychiatric Patients (TIC‐P),^42^ a validated patient‐reported outcome measure to capture utilization of health services that is not captured through linked government administration data.

#### 2.7.6 Intervention acceptability measures

Treatment Satisfaction Questionnaires (TSQ). This is a purpose‐built measure to assess the acceptability of online treatment courses and to measure participants' satisfaction with treatment.^32^

#### 2.7.7 Substudy outcome

We will conduct a brief survey of referring or primary health professionals about the usefulness of the intervention and care pathway, any consequences to their patient's management, and areas for improvement.

### 2.8 Data linkage

Participants will be asked to consent to have their data linked with their claims on the Medicare Benefits Schedule and Pharmaceutical Benefits Scheme and Public Hospital records to capture healthcare system costs including emergency department presentations, hospital admissions, nonadmitted care, medicines, primary care, and diagnostic services. Health record information will be obtained through the Australian Institute for Health and Welfare (AIHW), which is an Australian government service dedicated to secure linkage of health data for research purposes. The information received from AIHW will contain de‐identified data for all participants in a cohort.

### 2.9 Power and sample size

Sample size was determined in line with expert guidelines^43^ and calculations included data from a previous RCT, which observed a standard rate of symptom change on our primary outcomes of depression and anxiety of 24% each.^32^ We utilized specialized longitudinal software^44^ to account for our proposed generalized estimation equations models (GEE). Power analyses were conducted with Type I error set at α = 0.05 and Type II error at β = 80% and utilized a DELTA2 methodology.^43^

Our superiority analyses will compare the two active treatment groups with the TAU‐WLC. We calculated that this requires a sample size of 125:80 (e.g., unguided: control) to detect a difference in the rate of symptom change as small as 11% on the mental health primary outcomes from baseline to post‐treatment.^44^ Our noninferiority analyses will compare the unguided and guided treatment groups. In line with power estimation for non‐inferiority thresholds^44^ in mental health trials,^45^, ^46^ we considered a difference of 50% of the standard rate of symptom reduction in depression and anxiety symptoms (i.e., 12% reduction in symptoms at post‐treatment) as the prespecified margin of noninferiority.^47^ Based on these margins, we require a sample size of 125:125 (unguided: guided).

Utilizing a conservative attrition estimate of 20%, a final larger sample of 150:150:75 (“Guided: Unguided” control) was decided.

## 3 STATISTICAL ANALYSES AND REPORTING PLAN

### 3.1 Clinical efficacy

We will report clinical efficacy findings with and without data imputations under the intention‐to‐treat analysis and missing data assumptions (e.g., completer's analysis). The intention‐to‐treat approach will involve all outcomes being evaluated and imputed for all participants who were randomized and provide baseline data, consistent with previous research.^32^, ^46^

Clinical efficacy will be assessed utilizing longitudinal GEE models to assess changes in the primary and secondary clinical outcomes over time and between the three groups.

Post‐treatment is the primary endpoint for changes in the primary and secondary clinical outcomes. To determine whether post‐treatment improvements are maintained over time, within‐group time effects will be examined at 3‐ and 12‐month follow‐ups. For all analyses, the level of statistical significance will be set at alpha 0.05. All GEE models will include log‐link function and gamma scale parameters to account for the positive skew in the clinical outcome data.

### 3.2 Clinical significance

Clinical significance will be reported in several ways. Consistent with previous research,^32^ we will calculate the average percentage improvement (e.g. pre‐treatment mean score – post‐treatment mean score/pre‐treatment mean score) for each group from pre‐treatment to post‐treatment and 3‐ and 12‐month follow‐ups, for the clinical outcomes, using the estimated marginal means from the GEE models. Second, the proportion of participants achieving a clinical improvement (defined as ≥25%) and large clinical improvement (defined as ≥50%) on the primary outcomes will be calculated and compared between groups using generalized linear models using appropriate statistical models (e.g., GEE). In addition, deterioration (i.e., symptom increase at post‐treatment of ≥30% and within the clinical range) will be compared between groups. Based on these outcomes, the number needed to treat (NNT) will be calculated. Finally, Hedges *g* effect sizes will be calculated for the between‐group and within‐group effects.

### 3.3 Sensitivity analyses

Sensitivity analyses will be conducted for primary outcomes based on DSM‐5 diagnostic status and baseline symptom severity. Additionally, we may examine clinically important subgroups based on treatment adherence and any unbalanced or clinically important baseline characteristics.

### 3.4 Handling missing data

Following the principle of intention to treat, data from cases that provide baseline data but subsequently do not complete proceeding measures will be handled using a conditional missing at‐random assumption. Consistent with previous research, it is expected that higher baseline symptom severity on the primary outcomes and poorer treatment adherence will increase the likelihood of missing data.^45^, ^46^ Multiple imputations will be used to address missing cases in a conservative manner. This will involve missing outcomes being imputed from pools of participants with similar adherence levels and baseline characteristics (stratified), consistent with previous research.^32^ By employing this strategy, we aim to ensure that imputed data closely align with observed outcomes, enhancing the validity of our analyses.^45^

### 3.5 Health economic evaluation

As with similar programs,^29^ the costs associated with treatment outside the procedures are expected to be skewed to the right, with many participants potentially having zero or little healthcare costs while a small proportion of others having large healthcare costs. Heteroskedasticity within the data is also a potential problem. We will therefore employ a generalized linear model (GLM) to deal with skewness and heteroskedasticity.^48^

Healthcare resource use data for participants in each trial arm will be obtained from the Australian Institute of Health and Welfare (AIHW). Differences in healthcare costs will be compared to differences in health‐related quality of life measured using the ReQoL.^40^ Cost‐effectiveness will be reported as an incremental cost‐effectiveness ratio (ICER), which will be compared to an implicit threshold derived from recommendations made by the Australian Government's Pharmaceutical Benefits Advisory Committee.^49^ Uncertainty around the ICER will be explored using deterministic and probabilistic sensitivity analyses, which will be reported on a cost‐effectiveness acceptability curve.

We will assess the potential impact on government budgets from scaling up the most cost‐effective intervention within Australia. This will include estimating how many patients could benefit from the intervention and potential changes to the use of other services. We will develop a Health Economics Analysis Plan to guide the economic evaluation and align our modeling approach with the ISPOR Good Research Practices for cost‐effectiveness analysis.^50^ Reporting would adhere to the Consolidated Health Economic Evaluation Reporting Standards 2022^51^ and evaluation reporting methods.

### 3.6 Data security and handling, confidentiality, and security

Data will be collected using the eCentreClinic and Macquarie University's software systems, which are compliant with Australia's National Safety and Quality Digital Mental Health standard and Australian Standards for the storage of Health Data.

Research data will be stored in a restricted‐access SharePoint site for highly sensitive data. The investigators will have access to identified data to monitor participant progress/safety and allow for clinician support where relevant. No other investigators or external parties will be provided individually identifiable participant data and no de‐identified data will only be made available subject to approval from a HREC or Standards Committee.

### 3.7 Patient and public involvement

Patient and health professional advocacy organizations, Epilepsy Action Australia (EAA) and the Epilepsy Society of Australia (ESA), are partners of this grant. Representatives from these organizations, L.T. (EAA) and K.P. (ESA), have assisted with study design and will assist with patient recruitment.

## AUTHOR CONTRIBUTIONS

Dr Gandy and Prof Dear devised the study concept. Dr Gandy and Ms Wu wrote the original draft of the protocol. Dr Karin wrote the statistical aspects of the analysis. Prof Cutler and Ms Elizabeth Seil wrote the health economics aspects of the analyses. All authors contributed to revisions of the protocol.

## CONFLICT OF INTEREST STATEMENT

Dr Gandy, Prof Dear, and Prof Titov are the authors and developers of the Wellbeing Neuro Course but derive no personal or financial benefit from it. Prof Dear and Prof Titov are funded by the Australian Government to develop and provide a free national online assessment and treatment service, the MindSpot Clinic (www.mindspot.org.au), for adults with anxiety, depression, or chronic health conditions.

## ETHICAL APPROVAL AND PUBLICATION STATEMENT

The study has been granted ethics approval from the Human Research Ethics Committee of Macquarie University (No: 520231325151475). Results of this study will be disseminated through publication in peer‐reviewed journals and presentations at scientific conferences. Any amendments to the protocol will be approved by the HREC. These changes will also be updated on ANZCTR (trial number ACTRN12623001327673). We confirm that we have read the Journal's position on issues involved in ethical publication and affirm that this report is consistent with those guidelines.
